# Association of Oral Health with Risk of Rheumatoid Arthritis: A Nationwide Cohort Study

**DOI:** 10.3390/jpm13020340

**Published:** 2023-02-15

**Authors:** Yoonkyung Chang, Min Kyung Chung, Jung-Hyun Park, Tae-Jin Song

**Affiliations:** 1 Department of Neurology, Mokdong Hospital, College of Medicine, Ewha Womans University, Seoul 07985, Republic of Korea; 2 Division of Rheumatology, Department of Internal Medicine, College of Medicine, Ewha Womans University, Seoul 07804, Republic of Korea; 3 Department of Oral and Maxillofacial Surgery, Mokdong Hospital, College of Medicine, Ewha Womans University, Seoul 07985, Republic of Korea; 4 Department of Neurology, Seoul Hospital, College of Medicine, Ewha Womans University, Seoul 07804, Republic of Korea

**Keywords:** periodontitis, oral hygiene, rheumatoid arthritis toothbrushing, dental scaling

## Abstract

Periodontitis and rheumatoid arthritis (RA) are inflammatory diseases that share many similarities. We aimed to investigate the associations of periodontitis and oral hygiene status and behaviors with RA in a nationwide general population cohort. Participants from the National Health Screening cohort database of Korea who underwent oral health screening by dentists between 2003 and 2004 were included. The occurrence of RA was analyzed according to the presence of periodontitis, oral health examination findings, and behaviors. Overall, 2,239,586 participants were included. During a median of 16.7 years, RA occurred in 27,029 (1.2%) participants. The risk for incident RA was higher when participants had periodontitis (hazard ratio (HR) 1.2, 95% confidence interval (CI), 1.08−1.24) and an increased number of missing teeth (HR 1.5, 95% CI, 1.38−1.69). In contrast, better oral hygiene behaviors, such as a higher frequency of daily tooth brushing (HR 0.76, 95% CI 0.73–0.79, *p* for trend <0.001) and a recent history of dental scaling (HR 0.96, 95% CI 0.94–0.99), were associated with a lower occurrence of RA. Periodontitis and increased missing teeth were associated with an increased risk of RA. Maintaining good oral hygiene through frequent tooth brushing and regular dental scaling may reduce the risk of RA occurrence.

## 1. Introduction

Periodontal diseases are common inflammatory diseases affecting nearly 30% of the adult population worldwide [1]. Periodontitis, one of the periodontal diseases, is defined as inflammation of the tooth-supporting tissues and results in attachment loss and alveolar bone destruction [2]. Periodontitis starts with local inflammation in the oral cavity by colonizing pathogenic periodontal bacteria and with microbiota shift resulting from polymicrobial dysbiosis [3,4]. Dysbiosis can cause a host inflammatory response, thus leading to periodontitis and its consequences such as attachment loss and tooth loss [5]. It has been suggested that periodontitis can develop into various systemic disorders, including cancer, metabolic disorders, cardiovascular diseases, and neurological diseases [6,7], via unresolved hyperinflammation and disruption of the innate and adaptive immune system [6,8]. Furthermore, tooth loss is associated with an increased risk of various systemic diseases such as diabetes mellitus (DM), cardiovascular disease, certain cancers, and neurodegenerative diseases [9,10,11,12,13,14].

Rheumatoid arthritis (RA), which has a prevalence of 0.5% to 2% worldwide, is a chronic inflammatory disease characterized by synovial inflammation that results in joint damage [15]. RA is assumed to be triggered by various environmental factors such as smoking, infectious agents, obesity, and the microbiota in genetically susceptible individuals [16]. Among the environmental factors related to RA, increasing evidence has suggested that periodontitis might be one of the triggers [17,18]. 

Periodontitis and RA share several genetic and environmental risk factors. Genetic risk factors, such as shared epitopes within the β-chain of human leukocyte antigen (HLA) and tyrosine phosphatase (PTPN22), are involved in both diseases [19,20]. Smoking, which stimulates peptide citrullination by peptiylarginine deiminases (PADs), triggers an anticitrullinated peptide antibody (ACPA) response in RA and also worsens periodontitis [21,22]. Moreover, some periodontal bacteria, such as *Porphyromonas gingivalis* and *Aggregatibacter actinomycetemcomitans*, are suggested to play a crucial role in the link between periodontitis and RA [23].

Several epidemiologic studies have suggested a bidirectional relationship between periodontitis and RA. In US population data, patients with RA showed a 1.8 times increased risk of periodontitis [17]. A history of periodontitis was also associated with a mildly increased risk of RA in another population-based study [18]. Although there have been some cross-sectional studies demonstrating the association between periodontitis and RA [24,25], there have been few longitudinal studies that have evaluated if oral health conditions including periodontitis and oral-hygiene-related behaviors affect the incidence of RA in the general population. 

This study aimed to investigate the association of oral health examination estimates with the occurrence of RA with a null hypothesis of no association between oral health status or related behaviors and risk of RA in a nationwide population-based cohort database in a longitudinal setting. 

## 2. Materials and Methods

### 2.1. Data Source

This study used the National Health Insurance Service-National Health Screening (NHIS-HEALS) cohort database of Korea. The NHIS, controlled and supported by the Korean government, is the sole insurance provider in Korea and is administered to almost 97% of Koreans. The remaining 3% of the population is supported by the Medical Aid program administered by the NHIS [26,27,28]. People enrolled in the NHIS are encouraged to receive yearly standardized health screenings. The NIHS-HEALS cohort database contains the demographic data, socioeconomic status, and health screening information of individuals and a claims database consisting of diagnoses, prescriptions, and treatments. The health screening examination includes evaluation of height, weight, and blood pressure; laboratory tests; questionnaires on lifestyle such as oral hygiene behaviors; and oral health examinations performed by a dentist. Dentists examine participants for the number of lost teeth or other dental problems. Participants from the NHIS-HEALS were not involved in this study’s design, analysis, or report. This study was approved by the Institutional Review Board of Ewha Womans University College of Medicine (2020-08-018) and provided a waiver of consent.

### 2.2. Study Population

**Figure 1:**
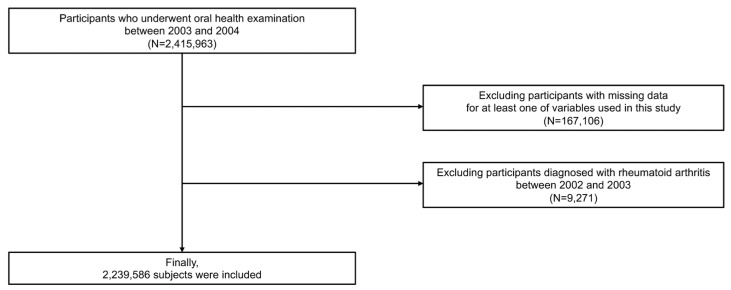
Flowchart of study subjects.

Our NIHS-HEALS cohort consisted of a total of 2,415,963 individuals aged 40−79 years who attended health examinations including oral health examinations from 2003 to 2004 (dataset number: NIHS-2022-01-313) [29,30,31]. Then, we excluded participants (*n* = 167,106) for whom data on at least one variable of need, including oral health examinations and laboratory findings, were missing. Next, participants (*n* = 9271) with a previous history of RA from 2002 (including a one-year washout period) to before the oral health examination were excluded. Finally, 2,239,586 participants were analyzed in this study (Figure 1).

### 2.3. Definitions and Variables

The index date was defined as the date of the oral health examination. The following baseline characteristics were identified on the index date: age, sex, household income, and body mass index. Information on smoking habits, alcohol consumption (frequency per week), and physical exercise (frequency per week) was collected using questionnaires. Smoking status was categorized as none, former, and/or current smoker. 

Periodontitis was defined as more than one claim including the diagnostics codes for periodontitis (International Classification of Diseases, 10th Revision (ICD)-10 codes: K052-K054) with at least one claim for one of the following procedure codes: U2232, U2240, U2233, U2221, U1010, U2211, U4454, U2222, U0010, U1051, U1052, U4455, U1060, U2231, M0111, and U4660 or a positive result on the oral health examination for a periodontal pocket detected by a dentist. Dentists also detected tooth loss during oral health examinations. The number of missing teeth was classified into four groups regardless of cause, such as periodontal disease or other dental reasons: 0, 1–7, 8–14, and over 15. Oral hygiene behavior was classified by the frequency of tooth brushing per day (0–1 time, 2 times, and ≥3 times) and by whether the participant received dental scaling at least once in the last year [11,32]. Comorbidities were identified according to the criteria (Appendix A) between January 2002 and the index date. We used ICD-10 when classifying diagnostic codes based on previous studies [9,10,11,12,13,14].

### 2.4. Study Outcomes

The outcome of our study was the occurrence of RA based on two or more claims of the ICD-10 diagnostic code M05 with the copayment beneficiaries’ program (V223) with the prescription of any disease-modifying antirheumatic drug [33]. This RA diagnostic algorithm was validated in a previous study (accuracy of more than 90%) [33]. The follow-up period was from the index date until RA occurred, the subject died, or December 2020, whichever occurred first.

### 2.5. Statistical Analysis

We used the chi-square test for categorical variables to compare the baseline characteristics and the independent *t*-test for continuous variables. Continuous variables are presented as the mean ± standard deviation, and categorical variables are presented as numbers (percentages). Kaplan–Meier survival curves with the log-rank test were used to evaluate the association of oral health status and oral hygiene behaviors with incident RA risk. The number of RA cases was divided by the sum of person-years to estimate the RA incidence. Cox’s proportional hazard regression was used to determine the association of oral health status and oral hygiene behaviors with the occurrence of RA, and hazard ratios (HRs) and 95% confidence intervals (CIs) were determined. A multivariable regression model adjusted for age, sex, household income, body mass index, smoking status, alcohol consumption, regular physical activity, and comorbidities (hypertension, DM, dyslipidemia, atrial fibrillation, and renal disease) was constructed. The number of lost teeth was divided into terciles and adjusted with no tooth loss as a reference. In consideration of the multicollinearity between oral hygiene and health behaviors, multivariable analysis was performed for each estimate regarding oral hygiene and oral health behaviors. Subgroup analyses for the association of the presence of periodontitis with the occurrence of RA were performed according to age, sex, and covariates. Regarding sensitivity analysis, a multivariable analysis was performed after excluding participants with RA within one year from the index date to minimize the possibility of reverse causality (landmark analysis). The assumption of the proportionality of hazards was tested using Shoenfeld’s residuals. No departure from the proportional hazards assumption was detected. All statistical analyses were performed using Statistical Analysis System software (SAS version 9.2, SAS Institute, Cary, NC). All values with *p*-values < 0.05 were considered statistically significant.

## 3. Results

Among a total of 2,239,586 participants, the average age of the included participants was 42.3 ± 12.8 years, and 66.4% were male. A total of 13,926 (0.6%) participants had more than 15 missing teeth, 923,237 (41.2%) participants brushed their teeth more than three times a day, and 512,683 (22.9%) participants had dental scaling within the previous year. The baseline participant characteristics and a comparative analysis according to the presence of periodontitis are shown in Table 1.

**Figure 2:**
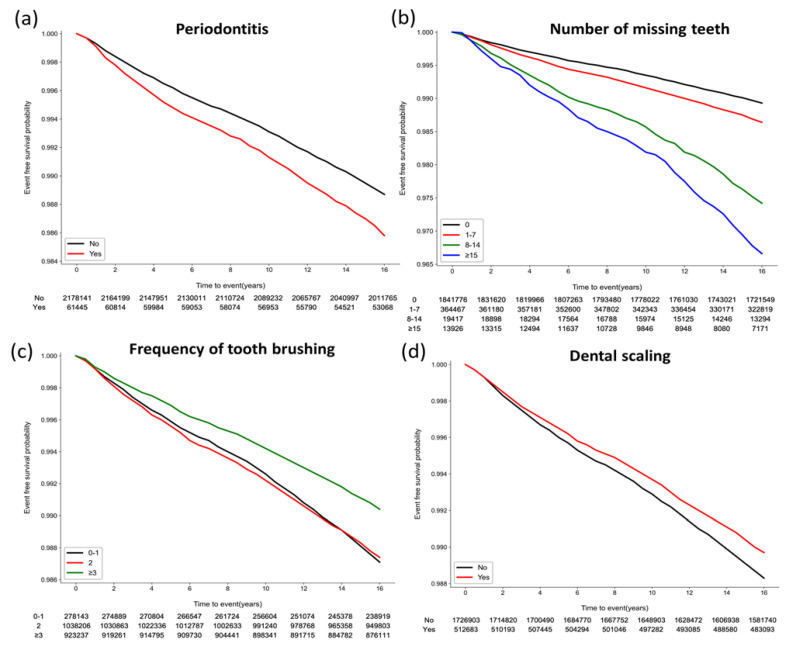
Kaplan–Meier survival curve associated with oral health status and oral hygiene behaviors for occurrence of rheumatoid arthritis. (**a**) Periodontitis (*p* < 0.001). (**b**) Number of missing teeth (*p* < 0.001). (**c**) Frequency of tooth brushing (times/per day) (*p* < 0.001). (**d**) Dental scaling within the previous year (*p* < 0.001).

During a median of 16.7 (interquartile range, 16.2–17.2) years, RA occurred in 27,029 (1.2%) participants. Figure 2 shows the Kaplan–Meier survival curves free from RA according to oral health status and oral hygiene behaviors. The risk for incident RA was higher when participants had periodontitis (*p* < 0.001) and an increased number of missing teeth (*p* < 0.001). Moreover, better oral hygiene behaviors including a higher frequency of daily tooth brushing and a history of dental scaling within the previous year were also associated with a lower occurrence of RA (*p* < 0.001) (Supplementary Table S1).

In the multivariable analysis, the presence of periodontitis was associated with the occurrence of RA (adjusted HR 1.16, 95% CI 1.08–1.24, *p* <0.0001). An increased number of missing teeth was associated with an increased risk of RA; the adjusted HR (in reference to subjects with no missing teeth) was 1.52 (95% CI 1.38–1.69, *p* < 0.001, *p* for trend < 0.001) for participants with more than 15 missing teeth. Furthermore, an increased frequency of tooth brushing was negatively correlated with the occurrence of RA. In reference to participants who brushed their teeth less than once a day, subjects who brushed their teeth more than three times a day (adjusted HR 0.76, 95% CI 0.73–0.79, *p* < 0.001, *p* for trend < 0.001) had a decreased risk of RA. Moreover, participants who received dental scaling within one year showed a significantly lower risk of occurrence of RA than those who did not (adjusted HR 0.96, 95% CI 0.94–0.99, *p* = 0.013) (Table 2). In the subgroup analysis, associations of periodontitis and oral health indicators with the occurrence of RA were consistently noted regardless of covariates (Supplementary Table S2). 

In the sensitivity analysis with a landmark analysis, the associations of periodontitis, number of missing teeth, frequency of tooth brushing, and dental scaling with the occurrence of RA were consistently noted even though the outcome (occurrence of RA) was redefined as RA after one year from the index date (Supplementary Table S3). 

## 4. Discussion

This study revealed that periodontitis and increased tooth loss were associated with an increased risk of seropositive RA and that maintaining better oral hygiene through frequent tooth brushing and dental scaling was associated with a decreased risk of RA. 

Several clinical, epidemiological, and serologic studies have shown a bidirectional association between periodontitis and RA [17,18,34]. Patients with RA were more likely to have periodontitis (OR 1.82, 95% CI 1.04–3.20) than the general population in a study using US national health data. [17] In a nationwide population-based study in Taiwan, patients with a history of periodontitis also had a higher risk of newly diagnosed RA than patients without a history of periodontitis [18]. Some systemic reviews have also demonstrated an association between periodontitis and RA [35,36,37]. Our result that patients with periodontitis had a longitudinally higher risk of RA is consistent with the findings of previous studies.

An increased number of missing teeth was a risk factor for RA occurrence in our study, which is similar to the findings of previous reports. Pablo et al. showed that the prevalence of tooth loss was higher in patients with RA, and the association with complete tooth loss was robust in patients with seropositive RA [17]. This association between RA and tooth loss was evident in younger adults [38]. Tooth loss is a consequence of complex causes including periodontal diseases, old age, smoking, socioeconomic status, comorbidities, and medication usage such as prednisolone usage, which are referred to as periodontal burden [39,40]. As the aggravation of periodontitis can potentiate the risk of tooth loss, our result indirectly supports the link between periodontitis and the occurrence of RA. Moreover, tooth loss can cause nutritional deficiencies due to chewing difficulty, which can indirectly influence the development of RA [41].

Although our study did not demonstrate the mechanism of the link between periodontitis or tooth loss and the development of RA, some basic research support this association. Some periodontal bacteria that are crucial in the development of periodontitis have been suggested to induce autoimmunity in RA. The detection of antibodies against periodontal pathogens and DNA from periodontal bacteria in serum and synovial fluid of patients with RA supports this idea [23,42]. Bacterial DNA from periodontal sites to the synovial fluid of patients with RA is transported via the bloodstream as free DNA [23]. A typical periodontal pathogen, *P. gingivalis*, is a unique anaerobe that can express PAD and produce citrullinated peptides, which trigger ACPAs. With their various virulence factors, local inflammation of the gingival mucosa and synovial membrane is exacerbated by proinflammatory host immune responses and by inhibition of the host’s protective mechanism due to citrullination and protease degradation [43]. As expected, *P. gingivalis* induced periodontitis and ACPA-associated RA in an experiment with rats [44]. In particular, our study limited the outcome to the occurrence of seropositive RA. As the association between periodontitis and RA has been demonstrated, especially in seropositive RA [17,45,46], our results reconfirmed this association. In addition to *P. gingivalis*, other periodontal pathogens such as *Treponema denticola* and *A. actinomycetemcomitans* have also been shown to be associated with RA and are being studied [47]. Although some studies showed no link between periodontitis and RA [38,48], it might be attributed to inaccuracy in defining RA or periodontitis.

Poor oral hygiene is a critical factor associated with periodontitis and tooth loss; thus, maintaining good oral hygiene through tooth brushing and frequent dental visits is associated with a reduced risk of periodontitis [49]. Recently, several studies have demonstrated that oral hygiene is related not only to local inflammation but also to various systemic diseases [9,12,13,50]. Improving oral hygiene behaviors such as frequent tooth brushing and dental scaling showed preventive effects against systemic diseases including hypertension, DM, and end-stage renal disease (ESRD) [11,26,51]. Frequent tooth brushing more than two times a day was associated with a lower risk of cardiovascular disease in patients with hypertension [13]. The risk of new-onset DM was attenuated with frequent tooth brushing in a nationwide study in Korea [27]. A national cohort study in Taiwan also showed that dental scaling was related to a lower risk of progression to ESRD in a dose-dependent manner [52].

Moreover, this association between oral care behaviors and disease was reported in patients with RA [53,54]. Khare et al. identified that a group that received nonsurgical periodontal therapy, such as scaling, root planning, and oral hygiene instruction, showed significant improvement in all RA parameters compared with a group that did not [53]. Nonsurgical periodontal therapy reduced prostaglandin E2 (PGE2), interleukin-6 (IL-6), and metalloproteinase-8 (MMP-8) levels in the gingival crevicular fluid of patients with RA, which infers that nonsurgical periodontal treatment could attenuate the inflammatory process [55]. In a nationwide study of a US population, patients diagnosed with RA had fewer regular dental visits than those without RA [17]. Our study findings are in accordance with those of these previous studies, and our study has the strength of demonstrating new information that better oral hygiene behaviors reduce the risk of developing RA. Unlike previous studies that identified an association between oral health behaviors and RA in a single center or a small group, this study is significant in that it found this association in a large national cohort. 

There are some limitations in our study. Firstly, the severity of periodontitis, such as the detailed attachment loss, could not be investigated due to the nature of the data. Secondly, our study design cannot suggest a causal relationship as a retrospective observational study. Although the severity of periodontitis was not described in every subject, our data based on the results of an oral health examination performed by a dentist could minimize the error. Furthermore, this study has strengths in that we used large-scale nationally representative data that have been tracked for a long time to elucidate the association of oral health and oral behaviors with the occurrence of RA. Our nationwide results provide unique evidence supporting the benefits of improving oral hygiene or behavior for RA prevention.

## 5. Conclusions

The presence of periodontitis and an increased number of missing teeth may be risk factors for the occurrence of RA. Better oral hygiene care including tooth brushing and dental scaling might be associated with a decreased risk of RA. Further studies supporting the causative relationship between oral hygiene and RA are needed. Understanding the underlying mechanisms of the inter-relationship between oral hygiene and RA might be a cornerstone in finding novel predictive markers of disease initiation in individuals at risk of RA.

## Figures and Tables

**Table 1:** Baseline characteristics of participants according to periodontitis.

Variable	Total	Periodontitis (-)	Periodontitis (+)	*p*-Value	Standardized Difference
No. of participants (%)	2,239,586	2,178,141 (97.3)	61,445 (2.7)		
Age, years	42.30 ± 12.77	42.13 ± 12.72	48.18 ± 13.11	<0.001	0.47
Sex				<0.001	0.09
Male	1,486,710 (66.4)	1,443,442 (66.3)	43,268 (70.4)		
Female	75,2876 (33.6)	734,699 (33.7)	18,177 (29.6)		
Body mass index (kg/m^2^)	23.55 ± 14.43	23.55 ± 14.61	23.80 ± 4.27	<0.001	0.02
Household income				<0.001	−0.05
Q1, lowest	586,108 (26.2)	568,304 (26.1)	17,804 (29.0)		
Q2	811,459 (36.2)	789,865 (36.3)	21,594 (35.1)		
Q3	585,974 (26.2)	570,645 (26.2)	15,329 (25.0)		
Q4, highest	256,045 (11.4)	249,327 (11.5)	6718 (10.9)		
Smoking status				<0.001	0.08
Never	1,254,341 (56.0)	1,222,277 (56.1)	32,064 (52.2)		
Former	240,702 (10.8)	233,633 (10.7)	7069 (11.5)		
Current	744,543 (33.2)	722,231 (33.2)	22,312 (36.3)		
Alcohol consumption (days/week)				<0.001	0.13
None	1,497,601 (66.9)	1,459,259 (67.0)	38,342 (62.4)		
1−4	683,708 (30.5)	663,955 (30.5)	19,753 (32.2)		
≥5	58,277 (2.6)	54,927 (2.5)	3350 (5.5)		
Regular physical activity (days/week)				<0.001	−0.03
None	1,166,099 (52.1)	1,132,327 (52.0)	33,772 (55.0)		
1−4	912,091 (40.7)	88,9517 (40.8)	22,574 (36.7)		
≥5	161,396 (7.2)	156,297 (7.2)	5099 (8.3)		
Comorbidities					
Hypertension	417,862 (18.7)	402,078 (18.5)	15,784 (25.7)	<0.001	0.17
Diabetes mellitus	169,278 (7.6)	161,760 (7.4)	7518 (12.2)	<0.001	0.16
Dyslipidemia	282,436 (12.6)	273,606 (12.6)	8830 (14.4)	<0.001	0.05
Atrial fibrillation	3,741 (0.2)	3,587 (0.2)	154 (0.3)	<0.001	0.02
Cancer	20,580 (0.9)	19,813 (0.9)	767 (1.3)	<0.001	0.03
Renal disease	11,188 (0.5)	10,755 (0.5)	433 (0.7)	<0.001	0.03
Oral health status					
Number of missing teeth				<0.001	0.35
0	1,841,776 (82.2)	1,800,326 (82.7)	41,450 (67.5)		
1−7	364,467 (16.3)	34,7095 (15.9)	17,372 (28.3)		
8−14	19,417 (0.9)	17,486 (0.8)	1931 (3.1)		
≥15	13,926 (0.6)	13,234 (0.6)	692 (1.1)		
Oral hygiene behaviors					
Frequency of tooth brushing (times/day)				<0.001	−0.20
0−1	278,143 (12.4)	267,593 (12.3)	10,550 (17.2)		
2	1,038,206 (46.4)	1,007,319 (46.3)	30,887 (50.3)		
≥3	923,237 (41.2)	903,229 (41.5)	20,008 (32.6)		
Dental scaling				<0.001	−0.11
No	1,726,903 (77.1)	1,676,892 (77.0)	50,011 (81.4)		
Yes	512,683 (22.9)	501,249 (23.0)	11,434 (18.6)		

*p*-value by chi-square test. Data are expressed as the mean ± standard deviation or *n* (%). Q: quartile.

**Table 2:** Association of oral health status and oral hygiene behaviors with the occurrence of rheumatoid arthritis.

	Number of Participants	Number of Events	Event Rate (%) (95% CI)	Person-Years	Incidence Rate (Per 1000 Person-Years)	Adjusted HR (95% CI)	*p*-Value
Oral health status							
Periodontitis							
No	2,178,141	26,124	1.20 (1.18, 1.21)	35,519,239.96	0.74	1 (reference)	
Yes	61,445	905	1.47 (1.38, 1.57)	981,934.40	0.92	1.16 (1.08, 1.24)	<0.001
Number of missing teeth							
0	1,841,776	20,930	1.14 (1.12, 1.15)	30,190,662.71	0.69	1 (reference)	
1−7	364,467	5219	1.43 (1.39, 1.47)	5,848,836.32	0.89	1.20 (1.17, 1.24)	<0.001
8−14	19,417	475	2.45 (2.23, 2.67)	281,567.14	1.69	1.49 (1.36, 1.63)	<0.001
≥15	13,926	405	2.91 (2.62, 3.19)	180,108.18	2.25	1.52 (1.38, 1.69)	<0.001
Oral hygiene behaviors							
Frequency of tooth brushing (times/day)							
0−1	278,143	3753	1.35 (1.31, 1.39)	4,403,805.12	0.85	1 (reference)	
2	1,038,206	13,899	1.34 (1.32, 1.36)	16,904,333.57	0.82	0.98 (0.95, 1.02)	0.300
≥3	923,237	9377	1.02 (1.00, 1.04)	15,193,035.67	0.62	0.76 (0.73, 0.79)	<0.001
Dental scaling							
No	1,726,903	21,398	1.24 (1.22, 1.26)	28,070,869.40	0.76	1 (reference)	
Yes	512,683	5631	1.10 (1.07, 1.13)	8,430,304.96	0.67	0.96 (0.94, 0.99)	0.013

The multivariable model was adjusted for sex, age, body mass index, income level, smoking, alcohol consumption, regular physical activity, hypertension, diabetes mellitus, dyslipidemia, atrial fibrillation, cancer, and renal disease. CI, confidence interval; HR, hazard ratio.

## Data Availability

The data used in this study are available in the National Health Insurance Service-National Health Screening Cohort (NHIS-HEALS) database, but restrictions apply to the public availability of these data used under license for the current study. Requests for access to the NHIS data can be made through the National Health Insurance Sharing Service homepage (http://nhiss.nhis.or.kr/bd/ab/bdaba021eng.do, accessed on 1 September 2020). For access to the database, a completed application form, research proposal, and application for approval from the institutional review board should be submitted to the inquiry committee of research support in the NHIS for review.

## Supplementary Materials

The following supporting information can be downloaded at: https://www.mdpi.com/article/10.3390/jpm13020340/s1, Table S1: Factors associated with the occurrence of rheumatoid arthritis; Table S2: Subgroup analysis for the association of periodontitis with the occurrence of rheumatoid arthritis according to demographics or comorbidities; Table S3: Association of oral health status and oral hygiene behaviors with the occurrence of rheumatoid arthritis (landmark analysis).

## Appendix A

We identified hypertension according to the following criteria: (1) prescription of any antihypertensive medication with at least one claim of the diagnostic codes I10–15, (2) two or more claims of the diagnostic codes I10–15, (3) blood pressure ≥140/90 mmHg (systolic/diastolic), or (4) self-reported hypertension in the questionnaire. DM was defined as meeting one of the following criteria: (1) prescription of an antidiabetic agent with at least one claim of the diagnostic codes E11–14, (2) ≥2 claims of the diagnostic codes E11–14, (3) fasting serum glucose level ≥ 7.0 mmol/L, or (4) self-reported DM in the questionnaire. Dyslipidemia was defined as satisfying one of the following criteria: (1) prescription of a dyslipidemia-related agent with at least one claim of the diagnostic code E78, (2) two or more claims of the diagnostic code E78, or (3) total cholesterol ≥ 240 mg/dL. Atrial fibrillation was defined as two or more claims of the diagnostic code I48. Cancer was defined as having at least one admission or three instances of the outpatient diagnostic codes C00–97 with a specific registration code of V027 or V193–4. Renal disease was defined as an estimated glomerular filtration rate less than 60 mL/min/1.73 m^2^ or ≥2 claims of the diagnostic codes N17–19, I12–13, E082, E102, E112, and E132 [9,10,11,12,13,14].
