# Supplementary material for: Association of Oral Health with Risk of Rheumatoid Arthritis: A Nationwide Cohort Study
Source: J Pers Med. 2023 Feb 15;13(2):340. doi: 10.3390/jpm13020340 (PMC9960411; doi:10.3390/jpm13020340)
Supplement: Supplementary file 1 [file jpm-13-00340-s001.zip › jpm-2212356-supplementary.pdf]

Table S1. Factors associated with the occurrence of rheumatoid arthritis

Variable	Crude HR (95% CI)	p-value	Adjusted HR (95% CI)	p-value
Age, years				
<65	1 (reference)		1 (reference)	
≥65	2.86 (2.76, 2.96)	<0.001	1.79 (1.72, 1.86)	<0.001
Sex				
Male	1 (reference)		1 (reference)	
Female	2.78 (2.72, 2.85)	<0.001	2.44 (2.36, 2.52)	<0.001
Body mass index (kg/m^2)	1.00 (1.00, 1.00)	0.082	1.00 (1.00, 1.00)	0.064
Household income				
Q1, highest	1 (reference)		1 (reference)	
Q2	0.75 (0.73, 0.77)	<0.001	0.97 (0.94, 1.00)	0.029
Q3	0.79 (0.77, 0.82)	<0.001	1.10 (1.07, 1.14)	<0.001
Q4, lowest	0.97 (0.94, 1.01)	0.192	1.22 (1.17, 1.27)	<0.001
Smoking				
Never	1 (reference)		1 (reference)	
Former	0.54 (0.52, 0.57)	<0.001	0.98 (0.94, 1.03)	0.499
Current	0.45 (0.44, 0.47)	<0.001	0.90 (0.87, 0.94)	<0.001
Alcohol consumption (days/week)				
None	1 (reference)		1 (reference)	
1-4	0.53 (0.51, 0.54)	<0.001	0.83 (0.81, 0.86)	<0.001
≥5	0.99 (0.92, 1.06)	0.690	1.38 (1.28, 1.49)	<0.001
Regular physical activity (days/week)				
None	1 (reference)		1 (reference)	
1-4	0.67 (0.65, 0.69)	<0.001	0.88 (0.86, 0.90)	<0.001
≥5	1.02 (0.98, 1.07)	0.348	1.04 (0.99, 1.08)	0.121
Comorbidities				
Hypertension	1.65 (1.61, 1.70)	<0.001	1.39 (1.35, 1.44)	<0.001
Diabetes mellitus	1.52 (1.46, 1.58)	<0.001	1.21 (1.16, 1.26)	<0.001
Dyslipidemia	1.47 (1.42, 1.51)	<0.001	1.18 (1.14, 1.22)	<0.001
Atrial fibrillation	1.87 (1.49 ,2.36)	<0.001	1.16 (0.92, 1.46)	0.211
Cancer	1.74 (1.57, 1.92)	<0.001	1.34 (1.22, 1.49)	<0.001
Renal disease	2.12 (1.88, 2.39)	<0.001	1.18 (1.04, 1.33)	0.010
Oral health status				
Periodontitis				
No	1 (reference)		1 (reference)	
Yes	1.23 (1.15, 1.32)	<0.001	1.16 (1.08, 1.24)	<0.001
Number of missing teeth				
0	1 (reference)		1 (reference)	
1-7	1.28 (1.24, 1.32)	<0.001	1.20 (1.17, 1.24)	<0.001
8-14	2.33 (2.13 ,2.55)	<0.001	1.49 (1.36, 1.63)	<0.001
≥15	3.07 (2.78, 3.38)	<0.001	1.52 (1.38,1.69)	<0.001
Oral hygiene behaviors				
Frequency of tooth brushing (times/day)				
0-1	1 (reference)		1 (reference)	
2	0.97 (0.94, 1.01)	0.130	0.98 (0.95, 1.02)	0.300
≥3	0.74 (0.71, 0.77)	<0.001	0.76 (0.73, 0.79)	<0.001
Dental scaling				
No	1 (reference)		1 (reference)	
Yes	0.88 (0.86, 0.91)	<0.001	0.96 (0.94, 0.99)	0.013

The multivariable model was adjusted for sex, age, body mass index, income levels, smoking, alcohol consumption, regular physical activity, hypertension, diabetes mellitus, dyslipidemia, atrial fibrillation, cancer, and renal disease.

HR, hazard ratio; CI, confidence interval; Q, quartile.

Table S2. Subgroup analysis for the association of periodontitis with the occurrence of rheumatoid arthritis according to demographics or comorbidities

Variable	Adjusted HR (95% CI)	p-value for interaction effect
Age, years		0.119
<65	1.18 (1.10, 1.28)	
≥65	1.01 (0.87, 1.17)	
Sex		0.059
Male	1.18 (1.07, 1.30)	
Female	1.12 (1.02, 1.23)	
Body mass index (kg/m^2)		0.426
<25	1.11 (1.02, 1.21)	
≥25	1.21 (1.09, 1.35)	
Household income		0.069
Q1, lowest	1.11 (0.98, 1.25)	
Q2	1.29 (1.15, 1.44)	
Q3	1.04 (0.90, 1.20)	
Q4, highest	1.16 (0.96, 1.39)	
Smoking		0.286
Never	1.15 (1.06, 1.24)	
Former	1.28 (1.03, 1.59)	
Current	1.13 (0.98, 1.31)	
Alcohol consumption (days/week)		0.562
None	1.19 (1.11, 1.29)	
1-4	1.03 (0.88, 1.21)	
≥5	1.09 (0.81, 1.46)	
Regular physical activity (days/week)		0.659
None	1.17 (1.07, 1.27)	
1−4	1.17 (1.03, 1.32)	
≥5	1.08 (0.87, 1.35)	
Comorbidities		
Hypertension		0.276
No	1.17 (1.08, 1.27)	
Yes	1.12 (1.00, 1.25)	
Diabetes mellitus		0.470
No	1.16 (1.08, 1.25)	
Yes	1.11 (0.94, 1.32)	
Dyslipidemia		0.103
No	1.20 (1.12, 1.29)	
Yes	0.95 (0.81, 1.12)	
Atrial fibrillation		0.865
No	1.16 (1.08, 1.24)	
Yes	1.32 (0.47, 3.71)	
Cancer		0.072
No	1.17 (1.09, 1.25)	
Yes	0.64 (0.33, 1.24)	
Renal disease		0.194
No	1.16 (1.09, 1.24)	
Yes	0.80 (0.39, 1.61)	

HR, hazard ratio; CI, confidence interval; Q, quartile.

Table S3. Association of oral health status and oral hygiene behaviors with the occurrence of rheumatoid arthritis (landmark analysis)

	Number of participants	Number of events	Event rate (%) (95% CI)	Person-years	Incidence rate (per 1000 person-years)	Adjusted HR (95% CI)	p-value
Oral health status							
Periodontitis							
No	2178141	25060	1.15 (1.14, 1.16)	33356641.45	0.75	1 (reference)	
Yes	61445	876	1.43 (1.33, 1.52)	920926.81	0.95	1.17 (1.10, 1.25)	<0.001
Number of missing teeth							
0	1841776	20092	1.09 (1.08, 1.11)	28361742.00	0.71	1 (reference)	
1−7	364467	5002	1.37 (1.33, 1.41)	5487185.98	0.91	1.20 (1.17, 1.24)	<0.001
8−14	19417	455	2.34 (2.13, 2.56)	262344.29	1.73	1.50 (1.36, 1.65)	<0.001
≥15	13926	387	2.78 (2.50, 3.06)	166295.99	2.33	1.53 (1.38, 1.70)	<0.001
Oral hygiene behaviors							
Frequency of tooth brushing (times/day)							
0−1	278143	3598	1.29 (1.25, 1.34)	4127690.67	0.87	1 (reference)	
2	1038206	13350	1.29 (1.26, 1.31)	15873999.08	0.84	0.98 (0.94, 1.02)	0.280
≥3	923237	8988	0.97 (0.95, 0.99)	14275878.52	0.63	0.76 (0.73, 0.79)	<0.001
Dental scaling							
No	1726903	20528	1.19 (1.17, 1.20)	26356296.55	0.78	1 (reference)	
Yes	512683	5408	1.05 (1.03, 1.08)	7921271.71	0.68	0.96 (0.94, 0.99)	0.016

The multivariable model was adjusted for sex, age, body mass index, income levels, smoking, alcohol consumption, regular physical activity, hypertension, diabetes mellitus, dyslipidemia, atrial fibrillation, cancer, and renal disease.
CI, confidence interval; HR, hazard ratio.
